# Adjusting trial results for biases in meta‐analysis: combining data‐based evidence on bias with detailed trial assessment

**DOI:** 10.1111/rssa.12485

**Published:** 2019-07-01

**Authors:** K. M. Rhodes, J. Savović, R. Elbers, H. E. Jones, J. P. T. Higgins, J. A. C. Sterne, N. J. Welton, R. M. Turner

**Affiliations:** ^1^ AstraZeneca Cambridge UK; ^2^ University of Cambridge UK; ^3^ University of Bristol UK; ^4^ University Hospitals Bristol National Health Service Foundation Trust UK; ^5^ University of Cambridge and University College London UK

**Keywords:** Bias, Elicitation, Meta‐analysis, Meta‐epidemiology, Randomized controlled trials

## Abstract

Flaws in the conduct of randomized trials can lead to biased estimation of the intervention effect. Methods for adjustment of within‐trial biases in meta‐analysis include the use of empirical evidence from an external collection of meta‐analyses, and the use of expert opinion informed by the assessment of detailed trial information. Our aim is to present methods to combine these two approaches to gain the advantages of both. We make use of the risk of bias information that is routinely available in Cochrane reviews, by obtaining empirical distributions for the bias associated with particular bias profiles (combinations of risk of bias judgements). We propose three methods: a formal combination of empirical evidence and opinion in a Bayesian analysis; asking experts to give an opinion on bias informed by both summary trial information and a bias distribution from the empirical evidence, either numerically or by selecting areas of the empirical distribution. The methods are demonstrated through application to two example binary outcome meta‐analyses. Bias distributions based on opinion informed by trial information alone were most dispersed on average, and those based on opinions obtained by selecting areas of the empirical distribution were narrowest. Although the three methods for combining empirical evidence with opinion vary in ease and speed of implementation, they yielded similar results in the two examples.

## 1 Introduction

In a meta‐analysis, results from a set of similar studies are combined to summarize evidence for a specific research question. Meta‐analyses are widely used in healthcare research, and policy decisions taken by organizations such as the UK National Institute for Health and Care Excellence often rely on results from relevant meta‐analyses. It is therefore important that meta‐analyses, and the systematic reviews from which they are derived, use methods that minimize bias.

Meta‐epidemiological studies of large numbers of meta‐analyses provide empirical evidence to suggest that aspects of trial design may lead to biased estimates of intervention effects. The recent ‘Risk of bias in evidence synthesis’ (ROBES) study examined the association between the risk of bias judgements from Cochrane reviews with intervention effect estimates in 228 binary outcome meta‐analyses with completed risk‐of‐bias tables (Savovic *et al*., 2017). Cochrane risk‐of‐bias tables include judgements of whether the risk of bias is low, high or unclear in relation to specific aspects of the trial methods. The results suggested that problems with randomization and lack of blinding are on average associated with a modest (approximately 10%) exaggeration of intervention effect estimates. There is also evidence that trial results that are based on subjectively assessed outcomes are more susceptible to bias.

Since high or unclear risk‐of‐bias judgements are associated with an exaggeration of intervention effect estimates, bias assessments should be incorporated in the analysis and interpretation of meta‐analyses. Currently it is unclear how to use the information that is provided in Cochrane risk‐of‐bias tables to reduce the effect of known biases on the meta‐analyses presented. Two methods have recently been proposed for adjusting the results of trials that are included in a meta‐analysis for expected biases: by reducing the weighting given to trials at high or unclear risk of bias, and correcting for average expected bias. Welton *et al*. (2009) used empirical evidence on bias from meta‐epidemiological studies, whereas Turner *et al*. (2009) used expert opinion based on assessment of detailed trial information. Adjustment using empirical evidence requires relatively little work to incorporate published empirical evidence and so is less time consuming than using elicited opinion. However, it relies on the strong assumption that biases in the new meta‐analysis are exchangeable with those in the meta‐epidemiological data. Further, we must allow for variation in bias across a collection of meta‐analyses, and this can lead to imprecise predictions of bias in a new trial in a new meta‐analysis, and hence imprecise effect estimates in a bias‐adjusted meta‐analysis. Bias adjustment based on opinion is tailored to the particular studies that are included in the new meta‐analysis and does not require the exchangeability assumption. However, elicitation of expert opinion on bias based on trial information alone can be difficult.

The objective of this research is to present methods in which the empirical data‐based approach that was proposed by Welton *et al*. (2009) and the opinion‐based approach that was proposed by Turner *et al*. (2009) can be combined to gain the advantages of both, while making use of the risk‐of‐bias information that is available in Cochrane reviews. The paper is organized as follows. We first describe two case‐study meta‐analyses. We then describe a generic framework for bias adjustment in a meta‐analysis, before showing how to inform the unknown bias parameters. Next, we briefly describe the existing methods to bias adjustment based on empirical data‐based evidence or opinion. We then propose three approaches to bias adjustment based on a combination of empirical evidence on bias and opinion. Each method is applied to the case‐study examples.

## 2 Motivating examples

We selected two case‐study meta‐analyses from the ROBES database (Savovic *et al*., 2017), chosen to include a variety of risk‐of‐bias judgements for sequence generation, allocation concealment and blinding. The first meta‐analysis A comprises 10 randomized controlled trials (RCTs) comparing intravenous immunoglobin with placebo or no treatment for preventing sepsis (one or more episodes) in preterm and/or low birth weight infants (Ohlsson and Lacy, 2004). The second meta‐analysis B comprises 25 RCTs comparing antidepressants against a placebo for treatment of depression in physically ill people (Rayner *et al*., 2010). The outcome was response to treatment at 6–8 weeks. For both meta‐analyses we judged the outcomes to be subjectively assessed.

**Figure 1:**
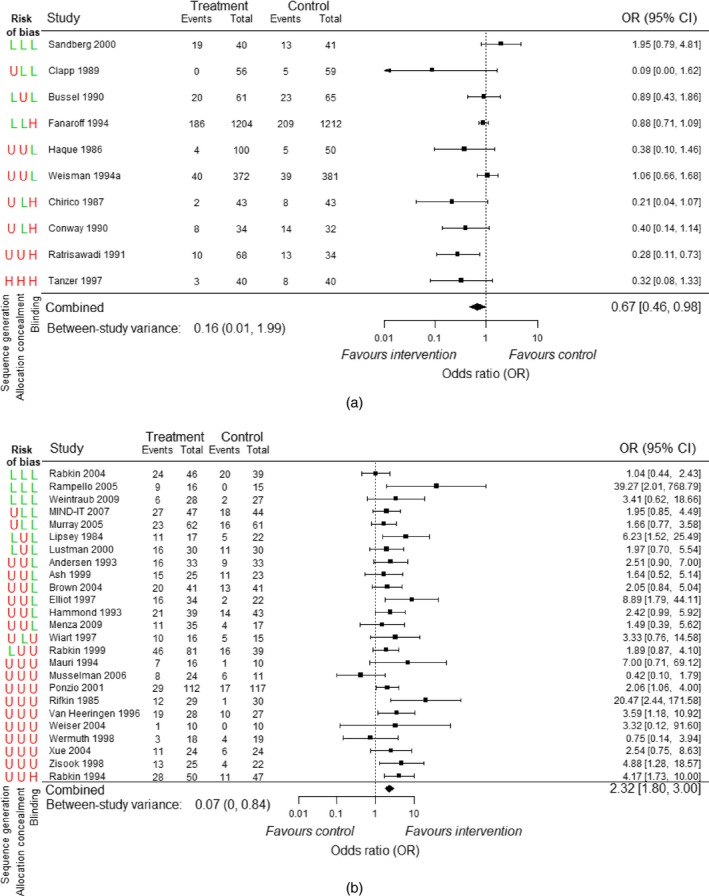
Trial results for case‐study meta‐analyses, with the risk of bias assessments for sequence generation, allocation concealment and blinding (H, U and L denote high, unclear and low risk of bias respectively; pooled intervention effects were estimated by using a random‐effects meta‐analysis model (DerSimonian and Laird 1986)): (a) meta‐analysis A (intravenous immunoglobin for prevention of sepsis): (b) meta‐analysis B (antidepressants for treatment of depression)

Fig. 1 shows forest plots of trial results for the two meta‐analyses, including indicators for whether the trials were judged to be at low, unclear or high risk of bias for sequence generation, allocation concealment and blinding. Throughout the paper we group the trials at high or unclear risk of bias because the proportion of trials with high risk of bias judgements is typically small (Savovic *et al*., 2012, 2017).

Risk‐of‐bias assessments for the trials that were included in the case‐study meta‐analyses were independently checked by co‐authors of this paper (Savović and Elbers), using the trial publications. Only a minority of trials were assessed to be at low risk of bias for sequence generation or allocation concealment. The number of trials that were assessed to be at low risk of bias for blinding was 5 (50%) for meta‐analysis A and 13 (52%) for meta‐analysis B. For both meta‐analysis A and meta‐analysis B, there were seven different bias profiles (combinations of high–unclear and low risk of bias judgements for sequence generation, allocation concealment and blinding). For example, the trial Clapp 1989 in meta‐analysis A was assessed to be at low risk of bias for allocation concealment and blinding but unclear risk of bias for sequence generation.

## 3 Bias adjustment model

Each trial in the meta‐analysis is assumed to provide an estimate of the underlying true intervention effect (which may include the effect of biases) and an appropriate distribution is chosen to allow for sampling error. The two case‐study meta‐analyses provide data in the form of binomial counts and we used a Bayesian hierarchical logistic regression model, assuming binomial within‐trial likelihoods. The trials at low risk of bias are assumed to provide an unbiased estimate *δ*_*i*_ of the true intervention effect (e.g. a log‐odds‐ratio for binary outcome data). We assume that these have a normal random‐effects distribution across trials:

$$\delta_{i}∼N\left(d,\tau^{2}\right).$$

The trials at high or unclear risk of bias are assumed to estimate the same intervention effect *δ*_*i*_ as the trials at low risk of bias plus a bias term *β*_*i*_ in trial *i*. We represent our knowledge about the likely amount of bias by a normal prior distribution for *β*_*i*_, with trial‐specific mean and variance:

$$\beta_{i}∼N\left(\mu_{i},\sigma_{i}^{2}\right).$$

We expect heterogeneity between trials to be partially explained by adjusting for biases (Rhodes *et al*., 2018), and we allow for unexplained heterogeneity *τ* ^2^ by using a random‐effects model for *δ*_*i*_.

This model differs from that proposed by Welton *et al*. (2009), since we do not specify a hierarchical prior structure for *β*_*i*_ and treat *μ*_*i*_ and $\sigma_{i}^{2}$ as fixed parameters.

We perform all analyses in a Bayesian framework. A vague *N*(0,10^5^) prior distribution was assigned to the average intervention effect *d*. We used a uniform(0,2) prior for the between‐trial standard deviation *τ*, which is sensible for analysis on the log‐odds‐ratio scale. Posterior estimates were obtained by using Markov chain Monte Carlo simulation implemented in the WinBUGS software (version 1.4.3) (Lunn *et al*., 2000). All the results that are presented were based on 500000 iterations, following a burn‐in period of 25000 iterations, which were sufficient to achieve convergence. Convergence was assessed according to the Brooks–Gelman–Rubin diagnostic tool (Brooks and Gelman, 1998), with three chains starting from widely dispersed initial values. The WinBUGS code for fitting the above bias adjustment model is given in section S1 of the on‐line supporting information.

## 4 Methods for quantifying bias

We describe five different approaches to deriving an informative prior distribution for the bias in each trial.

### 4.1 Method 1: data‐based approach

We constructed prior distributions for total bias *β*_*i*_ in each trial *i* in the new meta‐analysis by using empirical data‐based evidence from an external collection of meta‐analyses. Eligible meta‐analyses were those including trials at low risk of bias and trials at high or unclear risk of bias for each of the three bias components: sequence generation, allocation concealment and blinding, and reporting subjectively measured outcomes (so that they were as relevant as possible to the case‐study meta‐analyses). On the basis of an extension of ‘model 3’ of Welton *et al*. (2009), we used Bayesian hierarchical models to reanalyse data from 64 binary outcome meta‐analyses (866 trials) included in the ROBES database. The mathematical form of the model is given in section S2 of the on‐line supporting information. Although data were available on bias that is associated with incomplete outcome data, we chose not to consider this source of bias in our analyses, since the ROBES results showed no evidence of a change in intervention effect associated with risk of bias for incomplete outcome data (Savovic *et al*., 2017).

**Figure 2:**
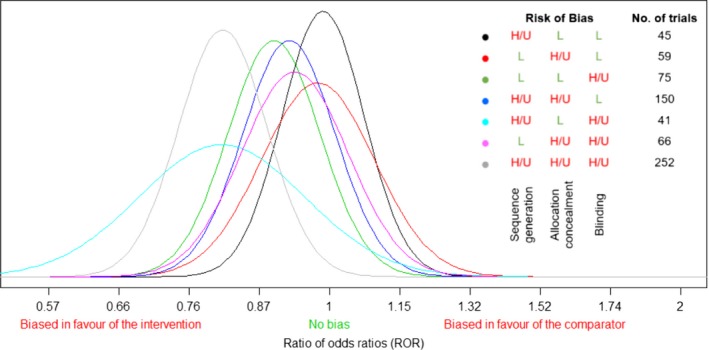
Data‐based distributions for the bias expected in a new trial, plotted on the log(ratio of odds ratios) scale and according to the trial's bias profile

For each bias profile, we derived an empirical predictive distribution for total bias *β*_new_ expected in a new trial with that profile. The derived predictive distributions for bias on the log(ratio of odds ratios) scale are displayed in Fig. 2 and represent the expected bias that is associated with a new trial having a specific bias profile rather than having low risk of bias judgements for sequence generation, allocation concealment and blinding. We shall refer to the predictive distribution for the relevant bias profile as the data‐based distribution for total bias *β*_*i*_ in a trial *i* in a new meta‐analysis with the same bias profile. This distribution serves as the $N(\mu_{i},\sigma_{i}^{2})$ prior distribution for total bias *β*_*i*_ in a bias adjustment meta‐analysis, where the model was described in the previous section.

### 4.2 Method 2: opinion‐based approach

As an alternative to using data‐based evidence, we consider an approach that was proposed by Turner *et al*. (2009). This approach constructs distributions for individual biases affecting a trial in a new meta‐analysis by using elicited opinion on the likely extent of bias, informed by a detailed assessment of the methodological quality of each trial. Experts are asked to summarize their beliefs about bias by providing a numerical range representing their uncertainty.

**Figure 3:**
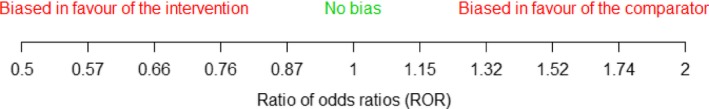
Elicitation scale for quantifying the extent of bias in a new trial, plotted on the log(ratio of odds ratios) scale

A number of bias assessors were given summary risk‐of‐bias information for trials in the new meta‐analysis and asked to mark an interquartile range IQR for total bias on the ratio of odds ratios scale (exp(*β*_*i*_)) in the new trial *i* relative to a trial at low risk of bias on an elicitation scale (Fig. 3), such that they expect the bias to be equally likely to lie inside or outside this range. The bias assessors were not provided with the data‐based distribution for the relevant bias profile (Fig. 2). We chose IQR for elicitation because it has been found that people perform better at assessing intervals corresponding to lower levels of certainty (O’Hagan *et al*., 2006). Bias assessors could write down a numerical range if they wished, and we made clear in the introductory training session that this could lie beyond the elicitation scale that was provided, to avoid anchoring effects (Johnson *et al*., 2010). Details of our elicitation process are provided in a later section.

Each elicited IQR on the ratio of odds ratios scale was rounded to the nearest 0.02 on the log(ratio of odds ratios) scale, mapped to a normal distribution for each trial *i*, and combined across assessors *j* as follows. We assumed that each IQR, on the log(ratio of odds ratios) scale, represented approximately *μ*_*ij*_ ± 0.67*σ*_*ij*_ as it would under normality and calculated values for *μ*_*ij*_ and *σ*_*ij*_. The pooled opinion‐based $N(\mu_{i},\sigma_{i}^{2})$ distribution for bias in each new trial was based on medians of the means *μ*_*ij*_ and medians of the standard deviations *σ*_*ij*_ of the assessors’ distributions, i.e. the medians of *μ*_*ij*_ and *σ*_*ij*_ across assessors *j* are the values that were used for *μ*_*i*_ and *σ*_*i*_ in the bias adjustment model that was described previously. Our method of pooling the opinion‐based bias distributions across assessors was chosen such that the pooled bias distribution would represent the opinion of a ‘typical’ assessor and not be influenced by extreme opinions (Turner *et al*., 2009). We chose this method in preference to linear pooling or logarithmic pooling, where the pooled opinion represents weakened or strengthened knowledge respectively in comparison with a single assessor (O’Hagan *et al*., 2006). We also quantify the consistency between assessor opinions in a later section.

### 4.3 Method 3: opinion‐based distributions combined statistically with data‐based distributions

The pooled opinion‐based distributions that were obtained in method 2 were statistically combined with the data‐based distributions in a Bayesian analysis. We treated the pooled opinion‐based distributions as data and the data‐based bias distributions as priors:

$$\begin{array}{cc} & \mu_{oi}∼N\left(\beta_{i},\sigma_{oi}^{2}\right),\\ & \beta_{i}∼N\left(\mu_{di},\sigma_{di}^{2}\right), \end{array}$$

where *μ*_o*i*_ and *σ*_o*i*_ are the mean and standard deviation of the pooled opinion‐based bias distribution for trial *i*, and *μ*_d*i*_ and *σ*_d*i*_ are the mean and standard deviation of the data‐based bias distribution.

Equivalently, we could have treated the pooled opinion‐based bias distributions as prior distributions and formally combined these with the data‐based distributions in a Bayesian analysis. Posterior estimates of total bias *β*_*i*_ in each trial *i* are used to inform the adjustment for bias in the meta‐analysis, which the data in the new meta‐analysis can also update.

This approach could be argued to be the fairest way to combine the empirical data‐based evidence and opinion, but assessors have previously indicated that quantifying the extent of bias that they expect in a trial is difficult (Turner *et al*., 2009). We therefore investigated alternatives in which we provided assessors with a data‐based evidence on the estimated amount of bias in trials with a similar risk of bias assessments in addition to summary risk‐of‐bias information for the new trial.

### 4.4 Method 4: numerical opinions informed by data‐based distributions

**Figure 4:**
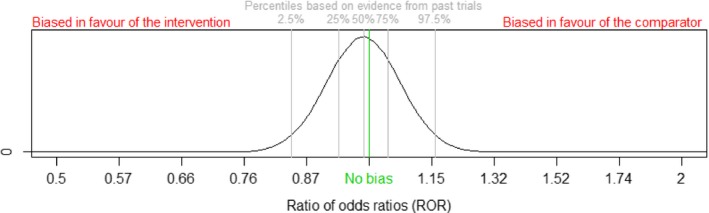
Data‐based bias distribution for elicitation of opinion in method 4

Method 4 is an alternative to the opinion‐based approach (method 2), where assessors are now provided with the relevant empirically derived bias distribution for each trial as well as summary trial information. By considering the characteristics of each new trial together with the data‐based bias distribution for a trial with the relevant bias profile, assessors were asked to mark on the *x*‐axis of the empirical bias distribution an IQR for total bias in each new trial (Fig. 4). Bias assessors could disagree with the data‐based evidence and provide a numerical range for bias that lies outside the range of the *x*‐axis. In essence, we ask the assessors to state where, among trials with the same bias profile as the trial at hand, this trial sits in terms of the magnitude of its bias. The ranges that were specified were used to form distributions for total bias in each new trial in the same way as in the opinion‐based approach, and we take the same approach to pooling distributions across assessors.

### 4.5 Method 5: opinions obtained by selecting areas from data‐based distributions

**Figure 5:**
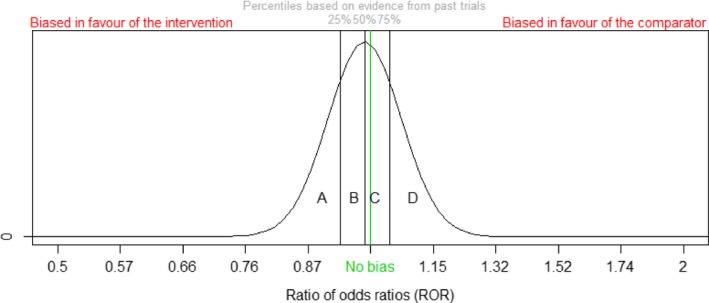
Data‐based bias distribution for elicitation of opinion in method 5

Our final method is similar to method 4. On the basis of the characteristics of each new trial, assessors were asked to select an area (e.g. *A*,* B*,* A* + *B*,* B* + *C* + *D*,* A* + *B* + *C* + *D*) of the empirical data‐based bias distribution for the relevant bias profile in which they expect the bias in the new trial to lie (Fig. 5). Assessors were asked to choose all areas in which they thought the bias might possibly lie, so that the area chosen represented their probability distribution for bias.

Each selected area of the empirical bias distribution is a truncated normal distribution. To map these to normal distributions for the bias in each new trial, we computed the 25% and 75% quartiles (i.e. an IQR) of the truncated distribution. Each IQR was used to form a normal distribution for total bias *β* in each new trial as in methods 2 and 4, which was then pooled across assessors in the same way as before.

## 5 Application to case‐study meta‐analyses

### 5.1 Elicitation process

We obtained opinions on the likely amount of bias in each trial during a 1‐day elicitation event separated into two sessions. It is recommended that opinion‐based prior distributions represent a breadth of opinions (Kadane, 1986). By increasing the number of assessors, it is more likely that the full range of opinions is covered, but this comes with increased costs. Conducting elicitations remotely would have been possible and would have avoided travel and subsistence costs, but face‐to‐face elicitations were preferred because experts can find the elicitation process difficult and it is useful to have facilitators on hand to answer questions (Chaloner and Rhame, 2001; Boobis *et al*., 2013). We recruited 12 review authors or methodologists to take part in this event to judge the extent of bias in trials included in two case‐study meta‐analyses. These assessors were well motivated and had sufficient knowledge to provide valid and reliable descriptions of their beliefs (Johnson *et al*., 2010). Invitation e‐mails were sent to review authors and methodologists, and to members of Cochrane Review groups. Potential assessors were eligible if they had detailed experience with the Cochrane risk‐of‐bias tool (Higgins *et al*., 2011), basic knowledge of statistics (e.g. understanding of a normal distribution, median, interquartile range, mean, standard deviation, odds ratios and confidence intervals), and understanding of and experience in conducting meta‐analyses. Assessors also had no conflicts of interest.

Opinions on the extent of total bias in each new trial were elicited by

- assessors marking their opinion on a scale (for methods 2 and 3);
- assessors marking their opinion on the data‐based bias distribution (for method 4) and
- assessors selecting an area of the data‐based bias distribution in which they expect the bias in the new trial to lie (for method 5).

Each elicitation strategy involved independent assessment of summary trial information, including our risk of bias judgements (low, high or unclear risk of bias for sequence generation; allocation concealment; blinding and incomplete outcome data) and supporting quotes from the original study references. Details on the trial design used, sample size, participants and outcomes were provided (see section S3 of the on‐line supporting information for an example trial information sheet). Although the ROBES database provided no empirical evidence of bias associated with incomplete outcome data (Savovic *et al*., 2017), assessors were provided with summary risk‐of‐bias information for this bias domain in each trial, which they could allow to influence their opinion. We chose not to provide the assessors with the original study references, to prevent the trial results from influencing assessor opinions.

The first elicitation session focused on strategy (a). At the start we gave an introductory presentation to the bias assessors, including details of the case‐study meta‐analyses, a practice elicitation exercise, discussion of the difference between uncertainty arising from imperfect knowledge and uncertainty arising from sampling variation, information on how we measure bias on the ratio of odds ratio scale and instructions on completing the forms for elicitation strategy (a). We are confident that assessors understood treatment effect sizes, interquartile ranges and the ratio of odds ratio quantity. We clarified any misunderstandings before beginning the elicitations.

The second session focused on strategies (b) and (c) in which we elicited opinion based on summary trial information in addition to empirical data‐based evidence on bias. This session began with an introduction to metaepidemiology and the ROBES database, summaries of the trials and meta‐analyses on which the empirical evidence on bias is based and instructions on completing the forms for elicitation strategies (b) and (c).

Each assessor assessed each trial once, using one of the three elicitation strategies. Each trial had at least one high–unclear risk of bias judgement (nine trials in meta‐analysis A and 22 trials in meta‐analysis B). The three strategies were assigned such that they were used an approximately equal number of times by each assessor. For each elicitation strategy, assessors were given a sample of 10 or 11 trials (three from meta‐analysis A and seven or eight from meta‐analysis B). Groups of four assessors used the same strategies for each trial. Trials were given to assessors in a random order by meta‐analysis; two assessors in each group assessed trials in meta‐analysis A first; the remaining two assessed trials in meta‐analysis B first. In total, each trial was assessed 12 times, with four independent assessments for each of the three elicitation strategies. Examples of each elicitation form are available in section S4 of the on‐line supporting information.

### 5.2 Descriptive analysis of elicitation data

Opinions on the extent of the total bias in each trial in meta‐analysis A and meta‐analysis B are displayed graphically in section S5 of the on‐line supporting information. We compare the opinions from the various elicitation strategies with caution, since the set of assessors is not the same for each trial and assessors may have different backgrounds and expertise. Opinions based on empirical bias distributions (elicitation strategy (b)) were usually less uncertain than those for strategy (a) where assessors did not have access to empirical evidence on bias. We note that no assessors disagreed with the empirical data‐based distribution (their opinions did not exceed the range of the *x*‐axis for the empirical distribution plotted). When choosing an area of the relevant empirical distribution (strategy (c)), most assessors chose at most two quartiles (50% of the distribution); 19/36 areas chosen by assessors represented 50% of the distribution and 11/36 areas represented 25% of the distribution. The majority of areas that were chosen were close to the median of the empirical bias distribution.

### 5.3 Consistency among assessors

We used intraclass correlation coefficients (ICCs) to investigate similarity of trial‐specific bias distributions across assessors for the three elicitation strategies. We fitted a mixed effects model to the means of the bias distributions, treating the trials as random effects and assessors as fixed effects (McGraw and Wong, 1996). The ICC was calculated as the proportion of total variation that is explained by between‐trial variation, and approximate 95% confidence intervals CI were based on a parametric bootstrapped distribution for the ICC derived from the mixed effects model.

In meta‐analysis A, the consistency between the assessors for total bias was low (ICC 16%; 95% CI 0–67%) for the plain elicitation scale (strategy (a)), meaning that only 16% of total variation in bias distributions could be explained by between‐trial variation rather than variation between assessors. Consistency between assessors was moderately high (ICC 65%; 95% CI 33–92%) for bias marked on the empirical bias distribution (strategy (b)), and also moderately high (ICC 53%; 95% CI 14–88%) for bias marked on a selected area of the empirical bias distribution (strategy (c)). The ICCs for meta‐analysis B were 19% (95% CI 0–47%), 79% (95% CI 65–90%) and 81% (95% CI 68–91%) for strategies (a), (b) and (c) respectively. The consistency between assessors is higher for meta‐analysis B, particularly in strategies (b) and (c) that used the empirical bias distributions. This is likely to be because 10/25 (40%) of trials in meta‐analysis B had the same risk of bias judgements (high–unclear risk of bias) for all three characteristics, and this combination of risk of bias judgements corresponded to a combination of the narrowest data‐based bias distributions.

Overall these results suggest that bias distributions from different assessors were much more consistent under strategies (b) and (c) than under strategy (a).

### 5.4 Comparison of trial‐specific bias distributions across methods

**Figure 6:**
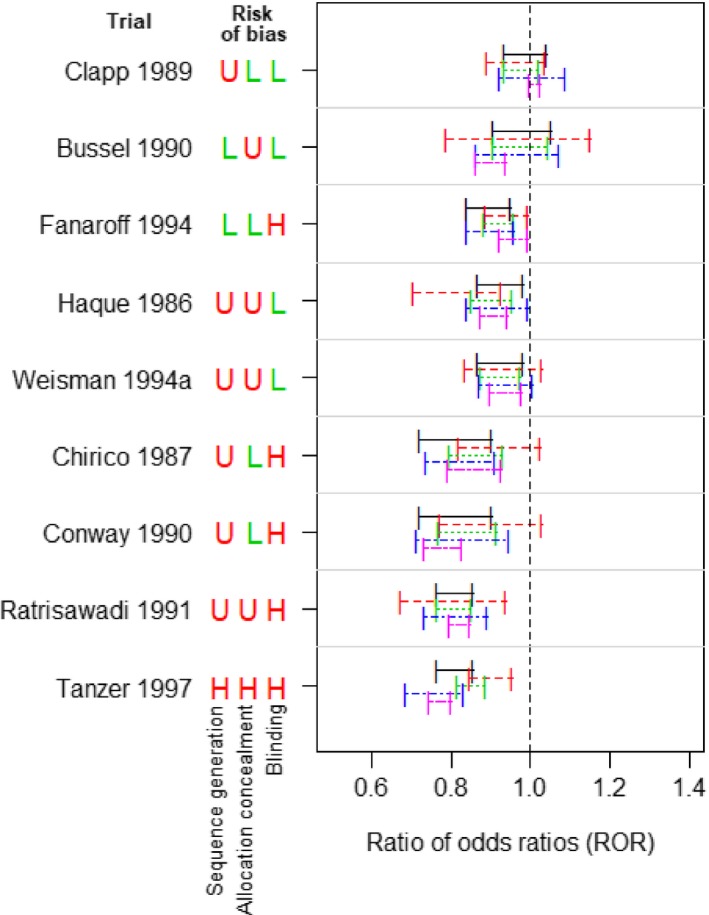
Meta‐analysis A: prior interquartile ranges of each bias distribution (H, U and L denote high, unclear and low risk of bias respectively): 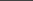, 1, data based; 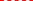, 2, opinion based; 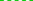, 3, opinion and data statistically combined; 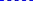, 4, data‐informed opinion (numerical); 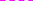, 5, data‐informed opinion (areas)

For each trial in the case‐study meta‐analyses we derived informative prior distributions using each of the five methods for quantifying bias. Across trials in meta‐analysis A, the median (interquartile range) for the average bias on the ratio of odds ratios scale was 0.85 (0.81–0.94) based on empirical evidence (method 1) and 0.90 (0.81–0.95) based on expert opinion (method 2). Summaries of average bias across trials obtained from methods 3, 4 and 5 were similar at 0.88 (0.84–0.94), 0.86 (0.80–0.94) and 0.87 (0.78–0.97). Trial‐specific bias distributions for meta‐analysis A are displayed in Fig. 6.

Across trials in meta‐analysis A, bias distributions were more dispersed on average for method 2 (opinion‐based, median standard deviation sd 0.16), followed by method 4 (numerical opinions informed by data‐based distributions, median sd 0.10), method 1 (data‐based, median sd 0.09) and method 3 (opinion and data statistically combined, median sd 0.08). Bias distributions for method 5 (opinion obtained by selecting areas of data‐based distribution) were narrower on average (median sd 0.06). The pooled opinion‐based bias distribution was wider than the data‐based distribution in all except two trials (Fanaroff 1994 and Chirico 1987), for which the two distributions were very similar in width. With the exception of Tanzer 1997, at high risk of bias for all three characteristics, the trial‐specific bias distributions were similar across methods (Fig. 6). Across all methods, biases in all except two trials (Clapp 1989 and Bussel 1990) were expected to favour the intervention on average. Bias distributions were narrower for the largest trial, Fanaroff 1994, and wider, on average, for Bussel 1990, with a single high or unclear risk of bias judgement for allocation concealment.

Across trials in meta‐analysis B, the median (interquartile range) for the average bias on the ratio of odds ratios scale was 0.81 (0.81–0.92) based on empirical evidence (method 1) and 0.85 (0.79–0.90) based on expert opinion (method 2). Summaries of average bias across trials obtained by using integrated methods 3, 4 and 5 were again similar at 0.82 (0.80–0.89), 0.81 (0.78–0.90) and 0.82 (0.80–0.92).

Trial‐specific bias distributions for meta‐analysis B are displayed in section S6 of the on‐line supporting information. Bias distributions were more dispersed on average for method 2 (median sd 0.20), followed by method 4 (median sd 0.14), method 1 (median sd 0.08) and method 3 (median sd 0.08). Bias distributions for method 5 were narrower on average (median sd 0.05).

### 5.5 Summary of feedback from assessors

We asked the assessors to provide feedback on our strategies for eliciting opinion on bias in a new trial. Responses were received from 11 of the 12 assessors. Table 1 summarizes the feedback. Six out of 11 assessors preferred to choose an area of the empirically derived distribution (elicitation strategy (c)) over marking an opinion on a plain scale or on the empirically derived distribution. Marking an opinion on a plain scale (strategy (a)) was given higher difficulty ratings by the assessors, whereas difficulty ratings given to strategies (b) and (c) were fairly similar. However, the number of assessors was very small, so these findings must be treated with caution.

**Table 1:** Feedback from assessors

*Question response*	*Number of assessors (%)*
*Question 1: what was your preferred elicitation strategy?*	
Mark opinion on scale based on trial information alone	3 (27%)
Mark opinion on empirically derived distribution	2 (18%)
Choose area of empirically derived distribution	6 (55%)
*Question 2: on a scale of 1 to 5, how difficult did you find marking opinion on bias on a scale?*	
Not at all difficult 1	2 (18%)
2	2 (18%)
Fair 3	2 (18%)
4	5 (45%)
Very difficult 5	0 (0%)
*Question 3: on a scale of 1*–*5, how difficult did you find marking opinion on the empirically derived distribution?*	
Not at all difficult 1	1 (9%)
2	4 (36%)
Fair 3	4 (36%)
4	1 (9%)
Very difficult 5	1 (9%)
*Question 4: on a scale of 1*–*5, how difficult did you find choosing an area of the empirically derived distribution?*	
Not at all difficult 1	3 (27%)
2	5 (45%)
Fair 3	2 (18%)
4	1 (9%)
Very difficult 5	0 (0%)

## 6 Meta‐analysis results

We compared results from six Bayesian meta‐analyses: ignoring bias (all trials taken at face value), and using each of the five sets of informative prior distributions from the five methods that were described above. Table 2 displays the estimated average intervention effect across trials and the estimated between‐trial heterogeneity variance *τ* ^2^ resulting from each method for meta‐analysis A, comparing intravenous immunoglobin against a placebo or no treatment for preventing sepsis (one or more episodes) in preterm and/or low birth weight infants, and meta‐analysis B, comparing antidepressants against a placebo for treatment of depression in physically ill people.

**Table 2:** Case‐study meta‐analysis A results, unadjusted for bias and by using five different approaches to bias adjustment: posterior medians; with 95% credible intervals in parentheses

*Method*	*Intervention effect*	*Heterogeneity*
	*(odds ratio)*	*varianceτ* ^2^
*Meta‐analysis A: outcome, sepsis, one or more episodes*		
Unadjusted Bayesian	0.58 (0.27–1.00)	0.48 (0.02–2.46)
Bias adjusted using method 1, data based	0.78 (0.35–1.22)	0.20 (0.001–2.07)
Bias adjusted using method 2, opinion based	0.67 (0.32–1.07)	0.33 (0.002–2.19)
Bias adjusted using method 3, opinions and data combined statistically	0.67 (0.32–1.07)	0.34 (0.005–2.13)
Bias adjusted using method 4, data‐informed opinions (numerical)	0.69 (0.33–1.09)	0.31 (0.003–2.10)
Bias adjusted using method 5, data‐	0.68 (0.33–1.07)	0.32 (0.002–2.14)
informed opinions (selected areas)		
*Meta‐analysis B: outcome, response to treatment (6–8 weeks)*		
Unadjusted Bayesian	2.56 (1.94–3.54)	0.12 (0.0005–0.73)
Bias adjusted using method 1, data based	2.30 (1.71–2.77)	0.07 (0.0002–0.63)
Bias adjusted using method 2, opinion based	2.24 (1.69–3.08)	0.08 (0.0002–0.67)
Bias adjusted using method 3, opinions and data combined statistically	2.29 (1.75–3.15)	0.10 (0.0003–0.70)
Bias adjusted using method 4, data‐ informed opinions (numerical)	2.26 (1.72–3.10)	0.09 (0.0001–0.68)
Bias adjusted using method 5, data‐informed opinions (selected areas)	2.28 (1.75–3.11)	0.08 (0.0002–0.64)

In meta‐analysis A, the results of an unadjusted Bayesian meta‐analysis show weak evidence that the intervention is effective; the estimate of the odds ratio OR is 0.58 (95% credible interval CrI 0.27–1.00). Adjusting for bias in the meta‐analysis by using empirical evidence leads to an estimate of the average odds ratio of 0.78 (95% CrI 0.35–1.22) which is closer to 1 (null value) compared with the model that ignores bias (OR 0.58; 95% CrI 0.27–1.00) (Table 2). The estimate of the intervention odds ratio also moves towards the null value 1 after adjusting for bias by using opinion alone (OR 0.67; 95% CrI 0.32–1.07). The three integrated approaches using combinations of empirical evidence and opinion to adjust for bias gave very similar results to those of the model using opinion alone. This is not surprising, given that the data‐based and opinion‐based distributions are quite similar (Fig. 6). Overall, there is little evidence for the effectiveness of intravenous immunoglobulin after adjustment for within‐study biases. After adjustment for bias based on empirical evidence, the estimated between‐trial heterogeneity decreases by 58%, compared with a reduction of approximately 33% when adjusting for bias by using opinion or a combination of empirical evidence and opinion.

In meta‐analysis B, the combined odds ratio comparing antidepressants with control is estimated as 2.56 (95% CrI 1.94–3.54) in an unadjusted Bayesian meta‐analysis. After adjusting for bias by using a combination of empirical evidence and opinion, the pooled intervention odds ratio is estimated as 2.29 (1.75–3.15) in method 3, 2.26 (1.72–3.10) in method 4 and 2.28 (1.75–3.11) in method 5. These estimates are very similar and lie between the pooled intervention effect estimates from the data‐based approach (OR 2.30; 95% CrI 1.71–2.77) and the opinion‐based approach (OR 2.24, 95% CrI 1.69–3.08) to bias adjustment in meta‐analysis. Heterogeneity variance decreases by 17% after adjusting for bias by using method 3. Reductions in between‐trial heterogeneity variance from adjusting for bias are similar irrespectively of the external evidence on bias that is used in the meta‐analysis. The evidence for the effectiveness of antidepressants remains strong after adjustment for within‐study biases.

The effect of bias adjustment on individual trial results in meta‐analysis A and meta‐analysis B is shown in section S7 of the on‐line supporting information. In meta‐analysis A, odds ratios less than 1 favour the intervention and so shift towards the null after bias adjustment because the external evidence suggests that bias is likely to favour the intervention. Odds ratios that are greater than 1 move further away from the null after bias adjustment in meta‐analysis A. In meta‐analysis B, odds ratios less than 1 favour the control and so move further away from the null after bias adjustment, whereas odds ratios that are greater than 1 shift towards the null. The widths of the credible intervals for the intervention odds ratios widen after adjusting for bias in meta‐analysis A but are not very much affected in meta‐analysis B because the estimates tend to be fairly imprecise anyway.

## 7 Discussion

In this paper we have proposed three new methods for adjusting the results of a meta‐analysis for the risk of bias in the trials included, by deriving informative prior distributions for total bias in a trial. The methods are based on combining empirical data with independent expert opinion, or using the empirical data to elicit expert opinions. Expert opinions that were informed by empirical data were considerably more consistent than those generated independently. In each of two example applications, our three new approaches to bias adjustment in meta‐analysis gave similar results, which were also similar to those obtained by using data‐based evidence alone or expert opinion alone.

In method 3, we elicit expert opinions independently from the empirical data and formally combine the resulting bias distributions by using Bayes's rule. This could be argued to be the most rigorous way to combine the two approaches, but it is more time consuming to implement and does not provide help to assessors by giving them the empirical distribution as a point of reference. It was therefore unsurprising that expert opinions were less consistent by using this approach than for methods 4 and 5. We gave equal importance to the data‐based and opinion‐based evidence, but others could choose to assign different weights to the two prior distributions for bias. In method 4, assessors choose numerical ranges for the total bias in the new trial by considering the specific characteristics of the new trial together with the empirical bias distribution. This approach is straightforward to implement since the elicited opinions directly provide distributions for the bias in each new trial. In method 5, assessors are asked to choose an area of the empirical bias distribution in which they expect the bias in the new trial to lie. This approach is arguably the simplest for assessors, but it is more time consuming for the analyst because the areas need to be mapped to distributions for the bias.

The elicitation strategy that was used in method 5 is more restrictive than those used in methods 3 and 4, since assessors are confined to selecting from a limited number of possible areas based on quartiles of the data‐based bias distribution. An alternative approach would be to ask the experts to build up a bias distribution by assigning weights or ‘chips’ to each quartile (or narrower intervals) of the data‐based bias distribution, to represent the probability of the bias lying in that quartile, in a similar way to Parmar *et al*. (1994).

The empirical data‐based approach makes the strong assumption that the trial‐specific biases in the new meta‐analysis are exchangeable with those in the meta‐epidemiological data that are used to provide empirical evidence. In our implementation of this approach, we restricted the meta‐epidemiological data used to subjectively measured outcomes (as in the case‐study meta‐analyses) and others could also restrict data to similar medical areas. In other settings, such restrictions may lead to prior information being based on small data sets with corresponding loss of precision.

In practice, the results of trials at low risk of bias for specific characteristics may be affected by other factors, for which the risk of bias has not been formally assessed. The advantage of using expert opinion on total bias, either alone or combined with data‐based evidence, is that the experts can allow all trial information that is provided to influence their opinion on total bias in a trial. We decided not to elicit opinion on trials at low risk of bias for the three characteristics that were assessed, to be consistent with adjustment using the data‐based approach which cannot account for biases within these trials. In practice we recommend that meta‐analysts consider using expert opinion to adjust results of trials with low risk of bias judgements for potential biases in meta‐analysis, although there would be no empirical evidence to use as a point of reference in the expert elicitation.

Our methods make use of risk‐of‐bias tables that are routinely created as part of Cochrane reviews and are commonly created also in non‐Cochrane reviews (Hopewell *et al*., 2013), meaning that the additional work of preparing summary trial information for the elicitation is minimal. However, the process of eliciting expert opinion is still time consuming and must be carried out for each trial in a meta‐analysis. Phillippo *et al*. (2018) have proposed a new method for network meta‐analysis that provides quantitative assessment of the effects of potential bias adjustments in each trial, by deriving bias adjustment thresholds which describe the smallest changes to the data that would result in a change to the overall conclusion of the meta‐analysis. By considering methods that were proposed by Phillippo *et al*. (2018), the elicitation process could potentially be reduced by focusing attention on the trials for which bias adjustment is likely to impact the meta‐analysis result.

Several other methods of adjusting for bias in meta‐analysis have been proposed. Eddy (1989), Wolpert and Mengersen (2004) and Greenland (2005) constructed separate models to represent the mechanism of each individual bias and made adjustments by incorporating these into the likelihood, or by using Monte Carlo simulation methods and approximate adjustments. These methods are difficult for non‐statisticians to implement and are therefore unlikely to be used in meta‐analyses in practice. Our methods are similar in intention but assume simpler forms for the trial‐specific biases. Although our implementation of bias‐adjusted meta‐analysis using Markov chain Monte Carlo methods within the WinBUGS software (Lunn *et al*., 2000) would be inaccessible to some researchers, an alternative two‐stage approach to implementation would enable the use of conventional meta‐analysis methods as used in Turner *et al*. (2009).

In this paper we focused on adjusting for biases within RCTs included in two binary outcome meta‐analyses. Both meta‐analyses included at least one trial at low risk of bias and covered many combinations of risk‐of‐bias judgements for the reported design characteristics of interest, but we note that this is not necessary for application of the methods. The results from different approaches to bias adjustment were similar in the two case‐study meta‐analyses, but they might differ in other examples. At present, there is a lack of empirical evidence on bias in meta‐analyses using other types of outcome data such as continuous or time‐to‐event data. Data‐based evidence on biases affecting non‐randomized studies is also sparse. An advantage of the opinion‐based approach is that it can be used to adjust for bias in meta‐analyses including both RCTs and non‐randomized studies. Once meta‐epidemiological data sets become available for other settings, our methods will be applicable to different types of outcome data and study designs.

A limitation is the accuracy of reported design characteristics. It is possible that well‐conducted trials could be poorly reported (Huwiler‐Muntener *et al*., 2002). Previous research suggests that many trials that were judged to be at unclear risk of bias for sequence generation and allocation concealment could be reclassified as being at low risk of bias if information that was not included in the trial publications was obtained (Hill *et al*., 2002; Vale *et al*., 2013). In this work, two co‐authors independently checked the risk‐of‐bias assessments for the trials that were included in case‐study meta‐analyses using the trial publications. Other researchers could request supporting information from the study authors to validate further against the Cochrane risk‐of‐bias judgements. Ideally, we would have carried out sensitivity analyses to assess the effect of changing the risk of bias from ‘unclear’ to ‘low’. However, in our meta‐epidemiological data set, trials at high risk of bias were sparse.

We cannot make strong recommendations on which method to use on the basis of application to example meta‐analyses. It is difficult to identify a potential study that could provide firm recommendations; simulation studies would not be possible with methods that use expert opinion. We suggest that the choice of combined method is based on the preferences of the systematic review authors, and that sensitivity analyses are conducted to assess the effect of the chosen method on the meta‐analysis results. When using any combined method (methods 3–5), we suggest the use of method 1 (the data‐based approach) in a sensitivity analysis. If method 3 (data and opinion statistically combined) is used, the results should also be compared with method 2 (opinion alone).

In summary, we proposed three new methods that allow adjustment for potential within‐trial biases in meta‐analysis, based on evidence from a large collection of published meta‐analyses and opinion informed by trial‐specific summaries. These methods make use of the risk‐of‐bias information that is available in Cochrane reviews. The three methods for combining empirical evidence with opinion vary in ease and speed of implementation but yielded similar results in two example applications.

## Supporting information

‘Supporting information’.

## References

[rssa12485-bib-0001] Boobis, A. , Flari, V. , Gosling, J. P. , Hart, A. , Craig, P. , Rushton, L. and Idahosa‐Taylor, E. (2013) Interpretation of the margin of exposure for genotoxic carcinogens—elicitation of expert knowledge about the form of the dose response curve at human relevant exposures. Food Chem. Toxicol., 57, 106–118. 23507349 10.1016/j.fct.2013.03.003

[rssa12485-bib-0002] Brooks, S. P. and Gelman, A. (1998) General methods for monitoring convergence of iterative simulations. J. Computnl Graph. Statist., 7, 434–455.

[rssa12485-bib-0003] Chaloner, K. and Rhame, F. S. (2001) Quantifying and documenting prior beliefs in clinical trials. Statist. Med., 20, 581–600. 10.1002/sim.694 11223902

[rssa12485-bib-0004] Eddy, D. M. (1989) The confidence profile method: a Bayesian method for assessing health technologies. Oper. Res., 37, 210–228. 10292450 10.1287/opre.37.2.210

[rssa12485-bib-0005] Greenland, S. (2005) Multiple‐bias modelling for analysis of observational data (with discussion). J. R. Statist. Soc. A, 168, 267–306.

[rssa12485-bib-0006] Higgins, J. P. T. , Altman, D. G. , Goetzsche, P. C. , Jueni, P. , Moher, D. , Oxman, A. D. , Savović, J. , Schulz, K. F. , Weeks, L. and Sterne, J. A. C. (2011) The Cochrane Collaboration's tool for assessing risk of bias in randomised trials. Br. Med. J., 343, article d5928. 22008217 10.1136/bmj.d5928 PMC3196245

[rssa12485-bib-0007] Hill, C. L. , LaValley, M. P. and Felson, T. (2002) Discrepancy between published report and actual conduct of randomized clinical trials. J. Clin. Epidem., 783–786. 10.1016/s0895-4356(02)00440-7 12384192

[rssa12485-bib-0008] Hopewell, S. , Boutron, I. , Altman, D. G. and Ravaud, P. (2013) Incorporation of assessments of risk of bias of primary studies in systematic reviews of randomised trials: a cross‐sectional study. Br. Med. J. Open, 3, article e003342. 10.1136/bmjopen-2013-003342 PMC3753473 23975265

[rssa12485-bib-0009] Huwiler‐Muntener, K. , Juni, P. , Junker, C. and Egger, M. (2002) Quality of reporting of randomized trials as a measure of methodologic quality. J. Am. Med. Ass., 287, 2801–2804. 10.1001/jama.287.21.2801 12038917

[rssa12485-bib-0010] Johnson, S. R. , Tomlinson, G. A. , Hawker, G. A. , Granton, J. T. and Feldman, B. M. (2010) Methods to elicit beliefs for Bayesian priors: a systematic review. J. Clin. Epidem., 63, 355–369. 10.1016/j.jclinepi.2009.06.003 19716263

[rssa12485-bib-0011] Kadane, J. B. (1986) Progress toward a more ethical method for clinical trials. J. Med. Phil., 11, 385–404. 10.1093/jmp/11.4.385 3819606

[rssa12485-bib-0012] Lunn, D. J. , Thomas, A. , Best, N. and Spiegelhalter, D. (2000) WinBUGS—a Bayesian modelling framework: concepts, structure, and extensibility. Statist. Comput., 10, 325–337.

[rssa12485-bib-0013] McGraw, K. O. and Wong, S. P. (1996) Forming inferences about some intraclass correlation coefficients. Psychol. Meth., 1, 30–46.

[rssa12485-bib-0014] O’Hagan, A. , Buck, C. , Daneshkhah, A. , Eiser, J. R. , Garthwaite, P. H. , Jenkinson, D. J. , Oakley, J. E. and Rakow, T. (2006) Uncertain Judgements: Eliciting Experts’ Probabilities. Chichester: Wiley.

[rssa12485-bib-0015] Ohlsson, A. and Lacy, J. (2004) Intravenous immunoglobulin for preventing infection in preterm and/or low birth weight infants. In *Cochrane Database of Systematic Reviews*, issue 1, article CD000361. Cochrane Collaboration. 10.1002/14651858.CD000361.pub2 14973955

[rssa12485-bib-0016] Parmar, M. K. , Spiegelhalter, D. J. and Freedman, L. S. (1994) The CHART trials: Bayesian design and monitoring in practice. Statist. Med., 13, 1297–1312. 10.1002/sim.4780131304 7973211

[rssa12485-bib-0017] Phillippo, D. M. , Dias, S. , Ades, A. E. , Didelez, V. and Welton, N. J. (2018) Sensitivity of treatment recommendations to bias in network meta‐analysis. J. R. Statist. Soc. A, 181, 843–867. 10.1111/rssa.12341 PMC6221150 30449954

[rssa12485-bib-0018] Rayner, L. , Price, A. , Evans, A. , Valsraj, K. , Higginson, I. J. and Hotopf, M. (2010) Antidepressants for depression in physically ill people. In *Cochrane Database of Systematic Reviews*, issue 3, article CD007503. Cochrane Collaboration. 10.1002/14651858.CD007503.pub2 PMC12279289 20238354

[rssa12485-bib-0019] Rhodes, K. M. , Turner, R. M. , Savović, J. , Jones, H. E. , Mawdsley, D. and Higgins, J. P. T. (2018) Between‐trial heterogeneity in meta‐analyses may be partially explained by reported design characteristics. J. Clin. Epidem., 95, 45–54. 10.1016/j.jclinepi.2017.11.025 PMC5828111 29217451

[rssa12485-bib-0020] Savović, J. , Jones, H. , Altman, D. , Harris, R. , Juni, P. , Pildal, J. , Als‐Nielsen, B. , Balk, E. , Gluud, C. , Gluud, L. , Ioannidis, J. , Schulz, K. , Beynon, R. , Welton, N. , Wood, L. , Moher, D. , Deeks, J. and Sterne, J. (2012) Influence of reported study design characteristics on intervention effect estimates from randomised controlled trials: combined analysis of meta‐epidemiological studies. Hlth Technol. Assessmnt, 16, 1–82. 10.3310/hta16350 22989478

[rssa12485-bib-0021] Savović, J. , Turner, R. M. , Mawdsley, D. , Jones, H. E. , Beynon, R. , Higgins, J. P. and Sterne, J. A. C. (2017) Association between risk‐of‐bias assessments and results of randomized trials in Cochrane reviews: the ROBES Meta‐Epidemiologic Study. Am. J. Epidem., 187, 1113–1122. 10.1093/aje/kwx344 PMC5928453 29126260

[rssa12485-bib-0022] Turner, R. M. , Spiegelhalter, D. J. , Smith, G. C. S. and Thompson, S. G. (2009) Bias modelling in evidence synthesis. J. R. Statist. Soc. A, 172, 21–47. 10.1111/j.1467-985X.2008.00547.x PMC2667303 19381328

[rssa12485-bib-0023] Vale, C. L. , Tierney, J. F. and Burdett, S. (2013) Can trial quality be reliably assessed from published reports of cancer trials: evaluation of risk of bias assessments in systematic reviews. Br. Med. J., 346, article f1798. 23610376 10.1136/bmj.f1798

[rssa12485-bib-0024] Welton, N. J. , Ades, A. E. , Carlin, J. B. , Altman, D. G. and Sterne, J. A. C. (2009) Models for potentially biased evidence in meta‐analysis using empirically based priors. J. R. Statist. Soc. A, 172, 119–136.

[rssa12485-bib-0025] Wolpert, R. L. and Mengersen, K. L. (2004) Adjusted likelihoods for synthesizing empirical evidence from studies that differ in quality and design: effects of environmental tobacco smoke. Statist. Sci., 19, 450–471.

