# Supplementary material for: Adjusting trial results for biases in meta‐analysis: combining data‐based evidence on bias with detailed trial assessment
Source: J R Stat Soc Ser A Stat Soc. 2019 Jul 1;183(1):193–209. doi: 10.1111/rssa.12485 (PMC6916311; doi:10.1111/rssa.12485)

Supporting information

S1 WinBUGS code for adjusting trial results for total bias in meta-analysis

#Bayesian meta-analysis of case study meta-analysis A dataset (Ohlsson and Lacy 2004), incorporating an informative prior for total bias in each trial at high or unclear risk of bias for sequence generation, allocation concealment and/or blinding.

```
model{
for (i in 1:10){
	rC[i]~dbin(pC[i],nC[i])  # Binomial distribution within trials (Control arm)
	rT[i]~dbin(pT[i],nT[i])  # Binomial distribution within trials (Treatment arm)
	logit(pC[i])<-alpha[i]
	logit(pT[i])<-alpha[i]+delta[i] + beta[i]*X[i]

	delta[i] ~dnorm(d,p.tau2)  #Random effects for intervention effect
	alpha[i]~dnorm(0,1.0E-5)  #Priors for baseline log-odds

	beta[i]~dnorm(mu[i],invsigma2[i])  #Trial-specific bias distribution
	invsigma2[i]<- 1/sigma2[i]
	sigma2[i]<- sigma[i]*sigma[i]

	s2[i]<-1/(nC[i]*pC[i])+1/(nC[i]*(1-pC[i]))+ 1/(nT[i]*pT[i])+1/(nT[i]*(1-pT[i]))
										# Approximate within-trial variance
	w[i]<-1/(s2[i]+tau2)
	pcw[i] <-(w[i]/sum(w[]))*100  #Percentage weight of trial in meta-analysis
	}
d ~ dnorm(0,1.0E-5)  #Prior for combined intervention effect (log odds ratio)
p.tau2<-1/tau2  #Prior for between-trial variance in intervention effect
tau2<-tau*tau
tau~dunif(0,2)
}
```

#Data from 10 trials included in case study meta-analysis A and parameters for trial-specific bias distributions derived from method 3. The trials are ordered as in Figure 1: 1=Sandberg 2000, 2=Clapp 1989, 3=Bussel 1990, 4=Fanaroff 1994, 5=Haque 1986, 6=Weisman 1994a, 7=Chirico 1987, 8=Conway 1990, 9=Ratrisawadi 1991, 10=Tanzer 1997.
#s, study; rC/nC, binary outcome data for control arm; rT/nT, binary outcome data for treatment arm; mu, mean of bias distribution; sigma, standard deviation of bias distribution; X, indicator of a high or unclear risk of bias judgement for at least one of sequence generation, allocation concealment and blinding.

```
s[]	rC[]	nC[]	rT[]	nT[]	mu[]	sigma[]	X[]
1	13	41	19	40	0	1	0
2	5	59	0	56	-.02195	.06566	1
3	23	65	20	61	-.02641	.1034	1
4	209	1212	186	1204	-.08356	.06108	1
5	5	50	4	100	-.103	.08294	1
6	39	381	40	372	-.07843	.07837	1
7	8	43	2	43	-.1493	.1153	1
8	14	32	8	34	-.1753	.1298	1
9	13	34	10	68	-.2134	.07974	1
10	8	40	3	40	-.1596	.06008	1
END
```

S2 Data-based evidence on bias

S2.1 Statistical analysis of the ROBES data

We modelled the estimated intervention effect θ_{im} (log odds ratio) in trial *i* of meta-analysis *m* as:

$$\delta_{im} + \gamma_{i1m}x_{i1m} + \gamma_{i2m}x_{i2m} + \gamma_{i3m}x_{i3m} + \gamma_{i4m}x_{i1m}x_{i2m} + \gamma_{i5m}x_{i1m}x_{i3m} + \gamma_{i6m}x_{i2m}x_{i3m} ,$$

where x_{ijm} are indicators of high or unclear risk of bias for design characteristic *j* (*j=1* for sequence generation, *j=2* for allocation concealment, *j=3* for blinding). Hence trials at low risk of bias for all three characteristics are assumed to estimate δ_{im}. We assume a normal random-effects distribution across trials for these intervention effects: $\delta_{im} \sim N(d_m, \tau_m^2)$. In the above equation, each γ_{ijm} (*j=1,2,3*) estimates the bias in intervention effect associated with a single high or unclear risk of bias judgement for characteristic *j* and low risk of bias judgements for the remaining characteristics in trial *i* of meta-analysis *m.*

A hierarchical model was fitted to the trial-specific biases, which allowed the main effects γ_{ijm} to vary within each meta-analysis *m* and mean bias b_{jm} to vary across meta-analyses *m*:

$$\gamma_{ijm} \sim N(b_{jm}, \kappa_j^2)$$
$$b_{jm} \sim N(b_{0j}, \phi_j^2).$$

The parameter κ_j estimates the average increase in between-trial heterogeneity among trials with a single high or unclear risk of bias judgement for design characteristic *j*, relative to those at low risk of bias for all three characteristics. The model assumes that average bias b_{jm} is exchangeable across meta-analyses *m* with mean b_{0j}, where exp(b_{0j}) is the average ratio of odds ratios (ROR) comparing the intervention effect in trials with one high or unclear risk of bias judgement for characteristic *j* to the intervention effect in trials with low risk of bias judgements for all three characteristics.

We allowed for interaction terms between pair-wise combinations of design characteristics. Interaction terms γ_{ijm} (*j=4, 5, 6*) were assumed to have the same two-level structure as the main effects γ_{ijm} (*j=1, 2, 3*), with distinct variance components. The average bias in trials with more than one high or unclear risk of bias judgement (on the log odds ratio scale) is estimated as the sum of the coefficients representing the effects of individual characteristics and the coefficients representing the

interaction terms involving these design characteristics. For example, for a trial at high or unclear risk of bias for sequence generation and allocation concealment and low risk of bias for blinding, the bias in intervention effect relative to a trial at low risk of bias for all characteristics is modelled as $\gamma_{i1m} + \gamma_{i2m} + \gamma_{i4m}$.

Within the full Bayesian model, we derived an empirical predictive distribution for total bias β_{new} expected in a new trial with each possible bias profile. For example, a predictive distribution for bias in a trial at high or unclear risk of bias for sequence generation and allocation concealment and low risk of bias for blinding is given by:

$$\beta_{new} \sim N(b_{01} + b_{02} + b_{04}, \kappa_1^2 + \kappa_2^2 + \kappa_4^2 + \phi_1^2 + \phi_2^2 + \phi_4^2).$$

The derived predictive distributions for bias may serve as empirically-based prior distributions for total bias β_{i} in each trial *i* in a new binary outcome meta-analysis, within the model for bias adjustment described in the section, “Adjusting for bias”.

S2.2 Descriptions of the meta-analyses and trials on which the distributions are based

64 meta-analyses (866 trials) from the ROBES database that assessed subjectively measured outcomes.

All meta-analyses include at least 5 trials and are informative for bias due to inadequate or unclear sequence generation, allocation concealment and blinding.

Distribution of outcome types

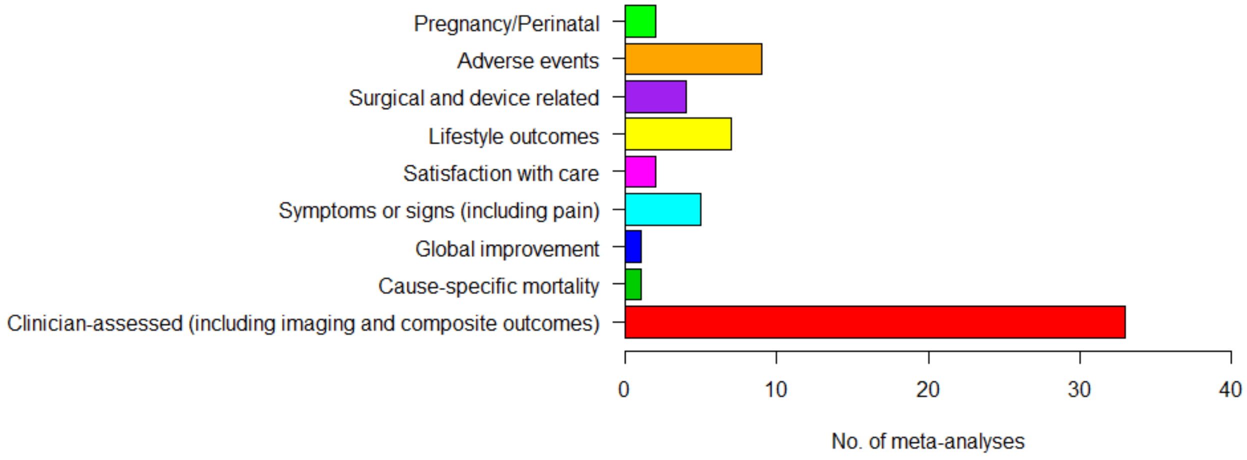

Proportion of trials at unclear risk of bias

Risk of bias			N	% unclear risk		
SG	AC	B		SG	AC	B
●	●	●	178	-	-	-
H/U	●	●	45	100%	-	-
●	H/U	●	59	-	95%	-
●	●	H/U	75	-	-	52%
H/U	H/U	●	150	98%	97%	-
H/U	●	H/U	41	100%	-	66%
●	H/U	○	66	-	86%	53%
H/U	H/U	H/U	252	92%	89%	68%

N, no. of trials with the risk of bias judgements
SG, Sequence generation; AC, Allocation concealment;
B, Blinding

H/U High/Unclear risk of bias

● Low risk of bias

S2.3 Empirical distributions for trial-specific biases

For each combination of risk of bias judgements (bias profile), we report a predictive normal distribution for the bias expected in a new trial with that same bias profile relative to a trial at low risk of bias. These distributions were derived from analysis of the 64 binary outcome meta-analyses (866 trials) included in the ROBES database.

Risk of bias			Bias distribution	Median ROR (95% CI)
SG	AC	B		
H/U	●	●	N(-0.013, 0.081^2)	0.99 (0.80 to 1.18)
●	H/U	●	N(-0.023, 0.111^2)	0.98 (0.73 to 1.15)
●	●	H/U	N(-0.110, 0.091^2)	0.90 (0.77 to 1.08)
H/U	H/U	●	N(-0.080, 0.091^2)	0.92 (0.78 to 1.11)
H/U	●	H/U	N(-0.213, 0.163^2)	0.81 (0.54 to 1.06)
●	H/U	H/U	N(-0.069, 0.105^2)	0.93 (0.75 to 1.13)
H/U	H/U	H/U	N(-0.211, 0.084^2)	0.81 (0.68 to 0.95)

SG, Sequence generation; AC, Allocation concealment; B, Blinding

H/U High/unclear risk of bias

● Low risk of bias

S3 Example summary trial information sheet

Ratrisawadi 1991 (Meta-analysis A: Intravenous immunoglobulin (pharmacological) versus placebo or no treatment)

Study characteristics

**Trial details**	Ratrisawadi 1991; Trial ID 11072
**Methods**	Randomised three arm parallel group trial
**Sample size**	N=102
**Participants**	**Experimental I:**<br>34 Infants (mean gestational age (SD) 31.5 (2.70) weeks; mean birth weight (SD) 1311.88 (145.52) g).<br>**Experimental II:**<br>34 Infants (mean gestational age (SD) 30.97 (2.18) weeks; mean birth weight (SD) 1330.31 (121.59) g).<br>**Control:**<br>34 Infants (mean gestational age (SD) 31.0 (1.98) weeks; mean birth weight (SD) 1290.14 (152.10) g).
**Interventions**	**Experimental I:**<br>Received 250 mg/kg of IVIG (Biotest Pharma, West Germany) within 4 hours of birth.<br>**Experimental II:**<br>Received 500 mg/kg of IVIG within 4 hours of birth.<br>**Control:**<br>Received no intervention.
**Outcome**	Sepsis (presence of clinical findings of sepsis plus positive blood cultures).
**Notes**	**Experimental group I and II were combined, and entered as <u>one experimental group in the meta-analysis</u>.**

Risk of bias assessment and summary of methods used (as described in paper)

**Adequate sequence generation?**<br>**<u>'Unclear' risk of bias</u>**	"The infants matched for gestational age, sex, weight and history of prolonged rupture of fetal membrane were randomly allocated into 3 groups of 34 each."<br><br>Groups were comparable at baseline.
**Allocation concealment?**<br>**<u>'Unclear' risk of bias</u>**	No details provided.
**Blinding?**<br>**<u>'High' risk of bias</u>**	No placebo was used.<br><br>"The drug was not given to group III (controlled group)."
Incomplete outcome data addressed?	Infants (number not stated) who expired within 24 hours of life or required blood exchange transfusion were excluded from the study. In spite of these exclusions the number of patients in each group is identical (N=34).<br><br>Denominator for analysis was the same as the number randomised per group.<br><br>'Unclear' risk of bias

S4 Example elicitation forms

S4.1 Elicitation strategy (i): assessor marks their opinion on a scale

Assessment Bussel 1990

L	**Sequence generation**	This trial has a low risk of bias judgement for sequence generation, a high/unclear risk of bias judgement for allocation concealment and a low risk of bias judgement for blinding. Where do you expect the ratio of odds ratios (ROR) comparing this trial against trials at low risk of bias to lie?
H/U	**Allocation concealment**	
L	**Blinding**	

Mark the expected ROR value by an X and the inter-quartile range (IQR) by two lines | on the axis below. Choose inter-quartile range limits such that you believe the true ROR is equally likely to lie inside rather than outside the range. Provide numerical values if you wish, which may fall outside the scale given.

Note that the axis is on the log ratio of odds ratios (log ROR) scale, to assist our analyses. We will round the ROR to the nearest 0.02 on the log ROR scale.

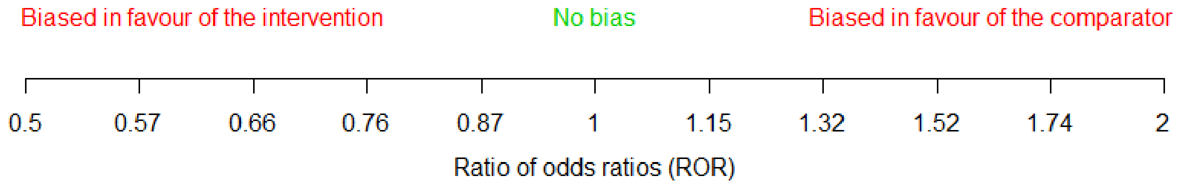

S4.2 Elicitation strategy (ii): assessor marks opinion on empirical bias distribution

Assessment Fanaroff 1994

L	Sequence generation	Evidence from a set of other trials at low risk of bias for sequence generation and allocation concealment and high/unclear risk of bias for blinding suggests that the bias might lie in the distribution represented by the Figure below.
L	Allocation concealment	
H/U	Blinding	

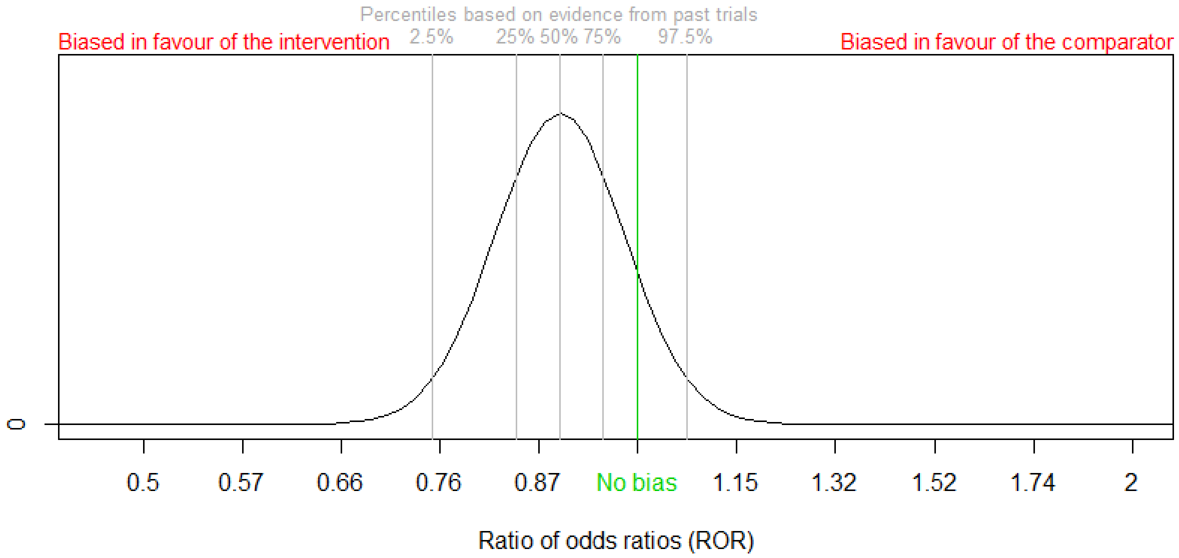

Given this information, where in this distribution do you expect the bias in this particular trial to lie?

Mark the expected ROR value by an X and the inter-quartile range (IQR) by two lines | on the x-axis below. Choose inter-quartile range limits such that you believe the true ROR is equally likely to lie inside rather than outside the range. Provide numerical values if you wish, which may fall outside the range of the x-axis. See below for an example.

Note that the x-axis is on the log ratio of odds ratios (log ROR) scale, to assist our analyses. We will round the ROR to the nearest 0.02 on the log ROR scale.

S4.3 Elicitation strategy (iii): assessor chooses area of empirical bias distribution

Assessment Weisman 1994a

H/U	**Sequence generation**	Evidence from a set of other trials at high/unclear risk of bias for sequence generation and allocation concealment and low risk of bias for blinding suggests that the bias might lie in the distribution represented by the Figure below.
H/U	**Allocation concealment**	
L	**Blinding**	

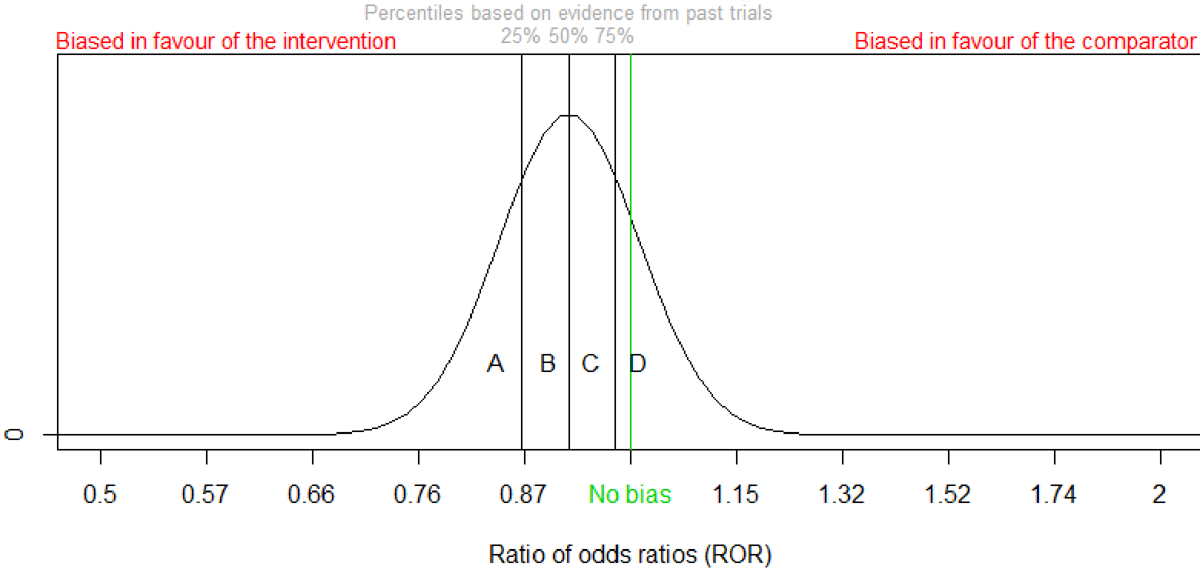

Given this information, where in this distribution do you expect the bias in this particular trial to lie? Choosing a larger area expresses greater uncertainty in your opinion.

	Area	Tick one option
More biased (overestimates treatment effect)	**A**	
	**A+B**	
	**A+B+C**	
	**B**	
About average bias for a trial with these risk of bias judgements	**B+C**	
	**B+C+D**	
	**C**	
	**C+D**	
Less biased/biased in opposite direction	**D**	
	**A+B+C+D**	

Figure S5a Meta-analysis A: opinions on the extent of bias, elicited from four out of twelve assessors, using three different methods. Plots (i) and (ii) display elicited inter-quartile ranges for bias, plot (iii) shows selected areas of empirically-derived bias distributions. H/U/L denote high/unclear/low risk of bias.

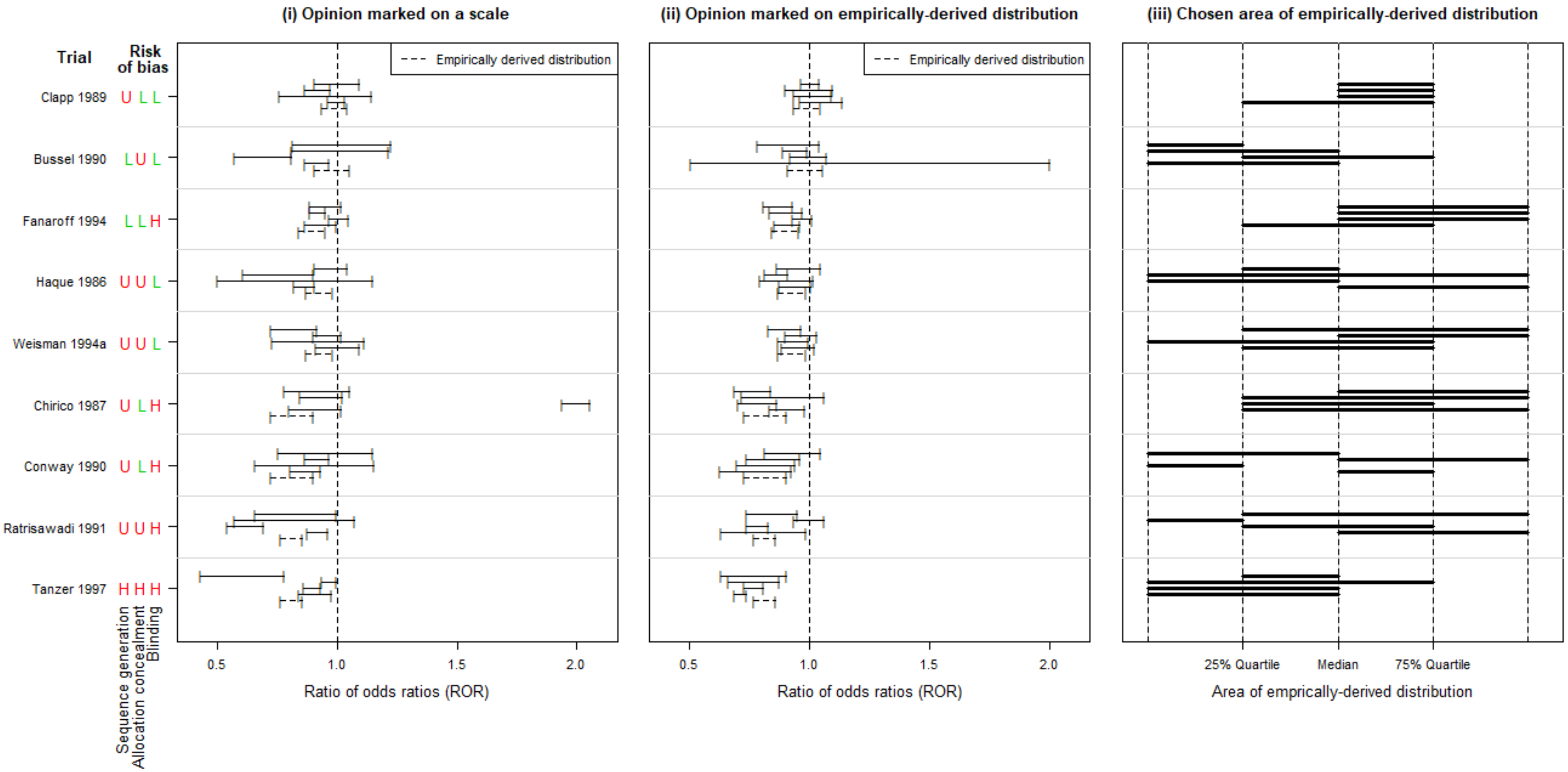

NB: The four assessors are not the same for each trial.

S5b Meta-analysis B: opinions on the extent of bias, elicited from four out of twelve assessors, using three different methods. The left and centre plots (i) and (ii) display elicited inter-quartile ranges for bias, the plot (iii) on the right shows selected areas of empirically-derived bias distributions. H/U/L denote high/unclear/low risk of bias.

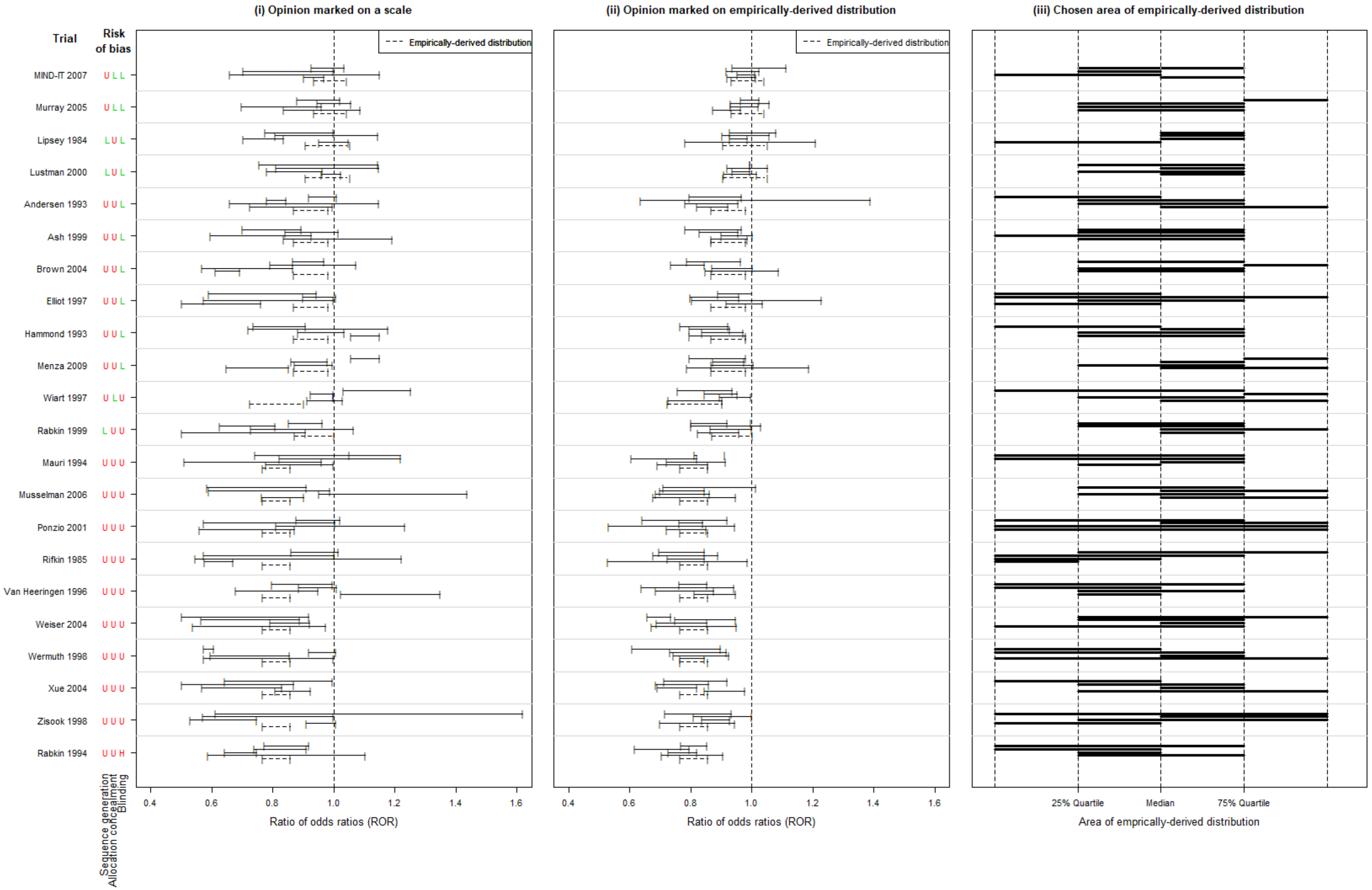

S6 Meta-analysis B: prior inter-quartile ranges of each bias distribution. H/U/L denote high/unclear/low risk of bias.

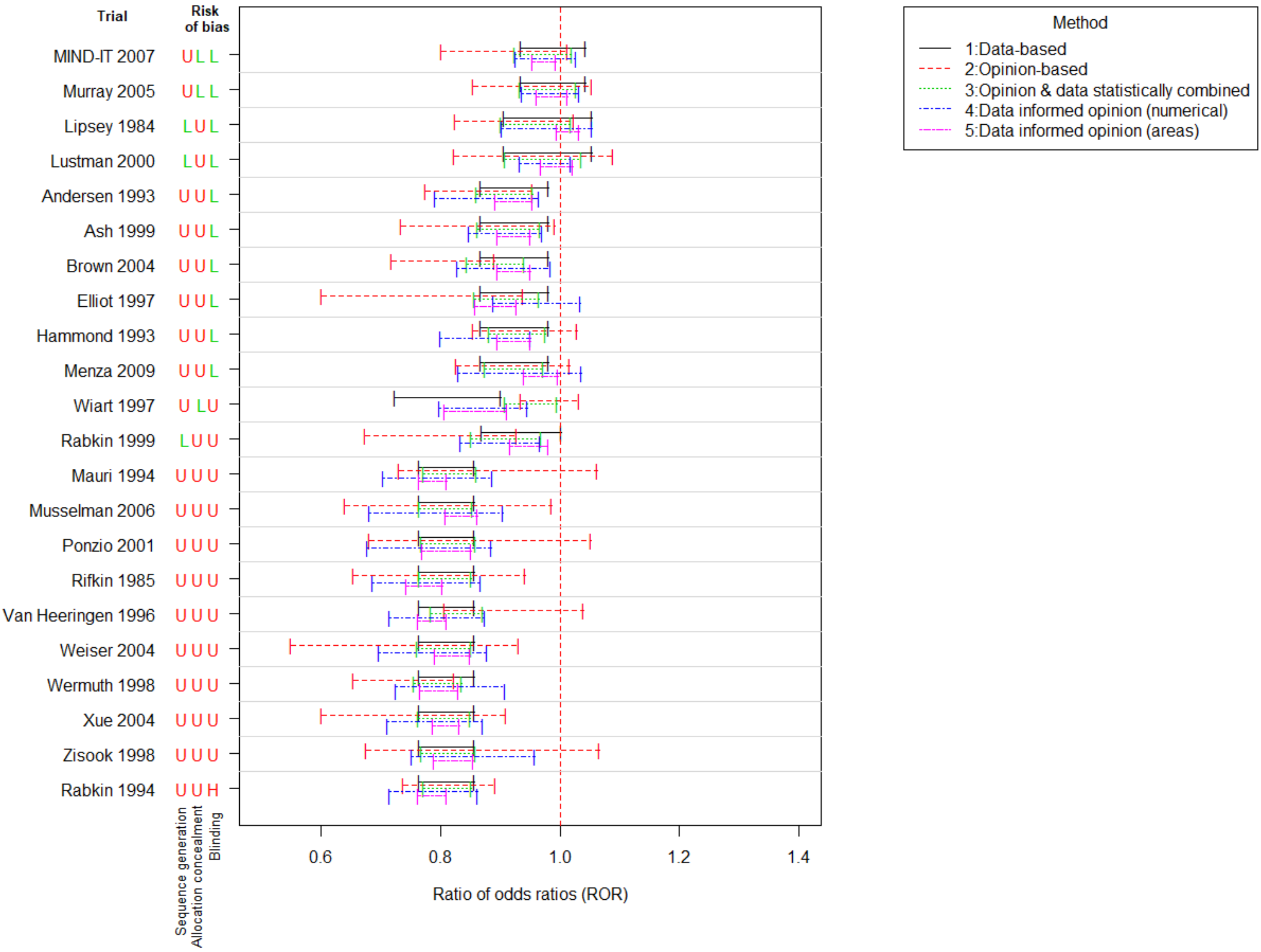

S7 Unadjusted and bias-adjusted trial results (odds ratios) for meta-analysis A, and the combined intervention effects estimated in a Bayesian random-effect meta-analysis as reported in Table 2. H/U/L denotes high/unclear/low risk of bias. Each individual trial result is the observed intervention effect adjusted for estimated bias. Weights are the inverse of the sum of the within-trial variance and the between-trial variance τ^2. Within-trial variances were calculated under the assumption of a normal approximation for the log odds ratio.

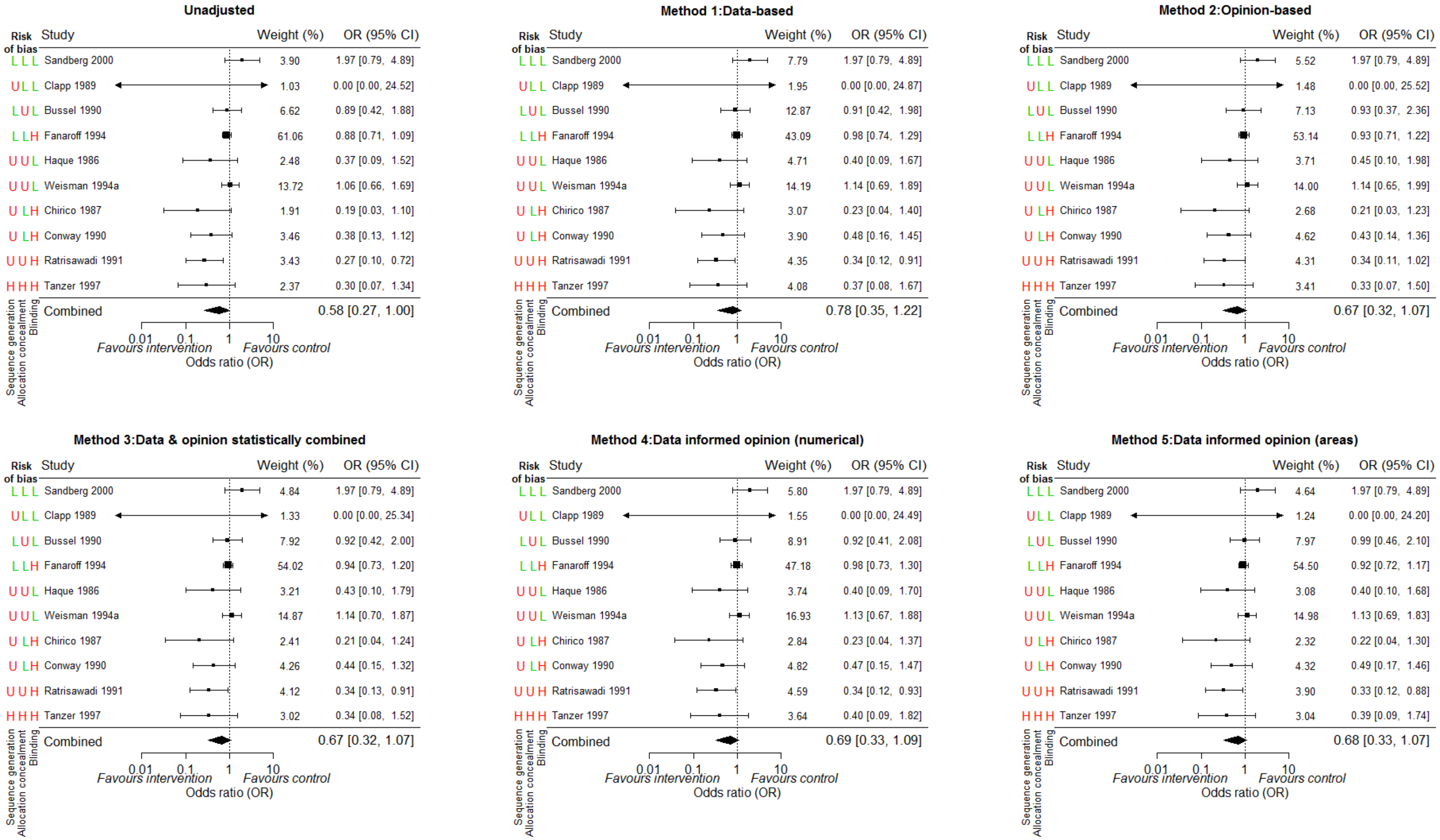

S8 Unadjusted and bias-adjusted trial results (odds ratios) for meta-analysis B, and combined intervention effects estimated in the Bayesian random-effects meta-analysis as reported in Table 2. H/U/L denote high/unclear/low risk of bias. Each individual trial result is the observed intervention effect adjusted for estimated bias. Weights are the inverse of the sum of the within-trial variance and the between-trial variance τ^2. Within-trial variances were calculated under the assumption of a normal approximation for the log odds ratio.

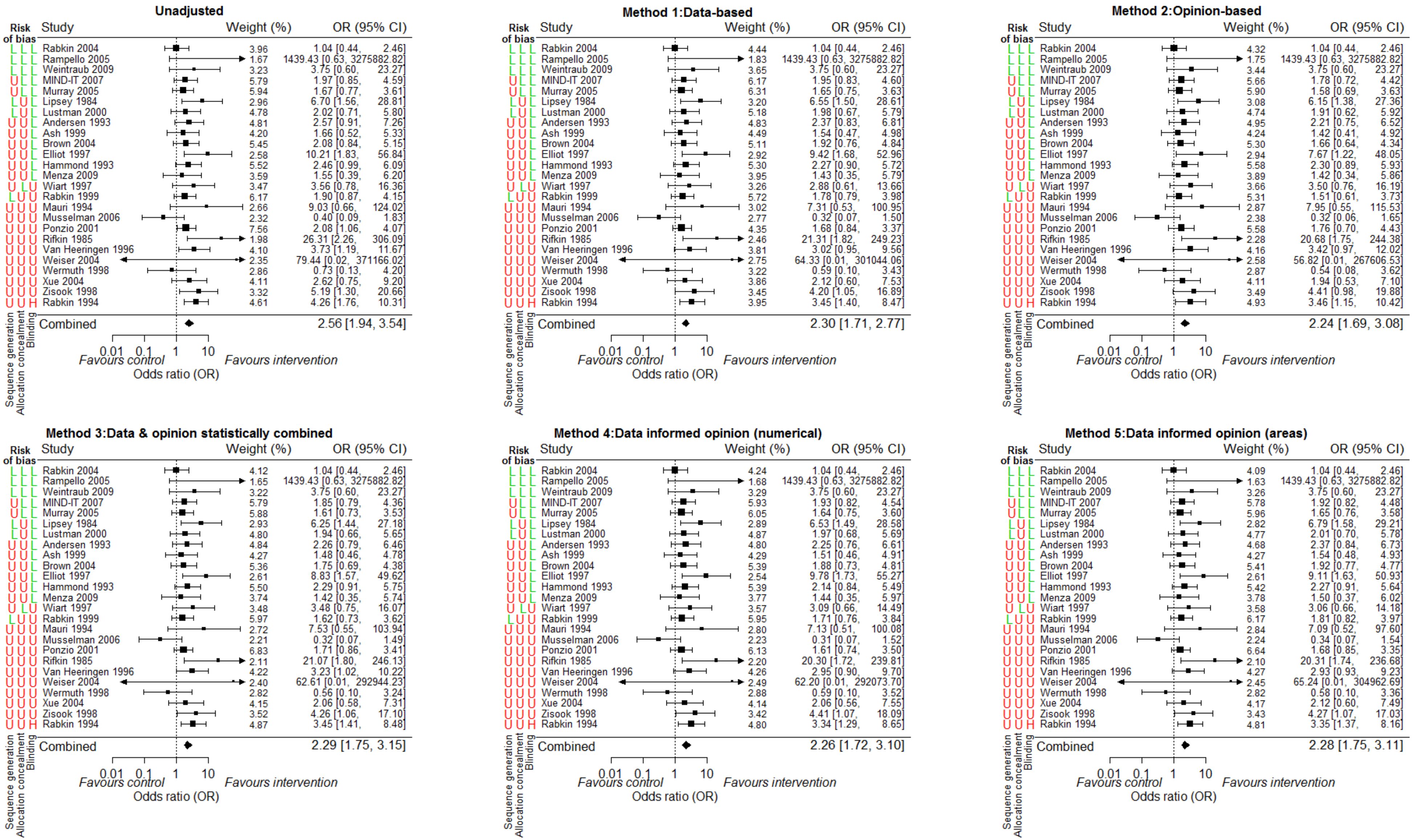

Supplement: Supplementary file 1 — ‘Supporting information’. [file RSSA-183-193-s001.pdf]
